# N^6^-methylation in the development, diagnosis, and treatment of gastric cancer

**DOI:** 10.2478/jtim-2023-0103

**Published:** 2024-03-21

**Authors:** Jiaxin Wang, Guiping Zhao, Yan Zhao, Zheng Zhao, Shuyue Yang, Anni Zhou, Peng Li, Shutian Zhang

**Affiliations:** Department of Gastroenterology, Beijing Friendship Hospital, Capital Medical University, Beijing, 100050, China

**Keywords:** gastric cancer, N^6^-methyladenosine, tumorigenesis, metastasis

## Abstract

Gastric cancer (GC) ranks third among cancers in terms of mortality rate worldwide. A clear understanding of the mechanisms underlying the genesis and progression of GC will contribute to clinical decision making. N^6^-methyladenosine (m^6^A) is the most abundant among diverse mRNA modification types and regulates multiple facets of RNA metabolism. In recent years, emerging studies have shown that m^6^A modifications are involved in gastric carcinoma tumorigenesis and progression and can potentially be valuable new prospects for diagnosis and prognosis. This article reviews the recent progress regarding m^6^A in GC.

## Background

Gastric cancer (GC) is an important malignant disease worldwide, ranking fifth in incidence and third in cancer-related deaths.^[ 1]^ The risk factors for GC include Helicobacter pylori (*H. pylori*) infection, cigarette smoking, alcohol consumption, and familial predisposition.^[2,3,4,5]^ The most common symptoms are dyspepsia, anorexia or early satiety, weight loss and abdominal pain.^[6]^ Endoscopic biopsy is used to histologically diagnose GC, and staging is performed using multiple methods, such as computed tomography (CT), endoscopic ultrasonography, positron emission tomography-CT (PET-CT) and laparoscopy. The choice of treatment is based primarily on the stage of the disease, the presence of biomarkers and the physician’s preferred option. Endoscopic resection is the preferred choice of treatment for early GC, while surgical resection, including total and subtotal gastrectomy, is currently the standard treatment for nonearly operable GC.^[7]^ Although surgery is the only curative treatment for GC, the addition of chemotherapy before (neoadjuvant), after (adjuvant) or in the perioperative period adds to the survival benefits. There are multiple alternatives for the treatment of metastatic GC, including cytotoxic monotherapy with first-line agents (antimetabolites, microtubule inhibitors, pyrimidine analogs) or combinations of two or three treatments.^[8]^ Presurgical chemotherapy treatment increases the chances of curative resection, eliminates early microscopic spread and allows *in vivo* response to treatment to be assessed.^[6]^ On the basis of the results of the phase II KEYNOTE-059 trial, pembrolizumab (Keytruda), a PD-L1 inhibitor, has been approved for the third-line treatment of GC.^[9]^ It is not currently recommended to add postoperative radiotherapy to perioperative or adjuvant chemotherapy.^[6]^ Immune checkpoint blockade has been established as a treatment for GC that has progressed after two or more lines of chemotherapy.^[6]^ Despite the multiple treatment options available to patients with GC, most patients succumb to the disease quickly due to the high degree of inter- and intratumor heterogeneity and the fact that most diagnoses occur at an advanced stage, at which point chemoresistance is common. In addition, patients often experience toxic side effects of chemotherapy such as nausea and vomiting, diarrhea and bone marrow suppression, which can affect the treatment effect and even lead to interruption of chemotherapy. In regard to immunotherapy, due to the spatial and temporal heterogeneity of PD-L1 expression and tumor mutational load, there is no universal standard for immunotherapy in GC. Further research into the function of the immune system in the development and progression of GC is needed.^[10]^ Furthermore, a large proportion of patients remain non-responsive to immunotherapy and more insight is needed into the complexities of the immune microenvironment in gastric cancer. Additional biomarkers also need to be explored to better identify subgroups of gastric cancer more sensitive to immunotherapy.^[11]^ Despite the decline in morbidity and mortality rates, GC accounted for over 1,000,000 new cases and 768,793 estimated deaths in 2020,^[1]^ and more GC cases are expected due to the aging population. Thus, it is urgent to elucidate the underlying mechanism related to tumorigenesis and progression to promote early diagnosis and improve prognosis.

## Introduction to m^6^A

**Figure 1:**
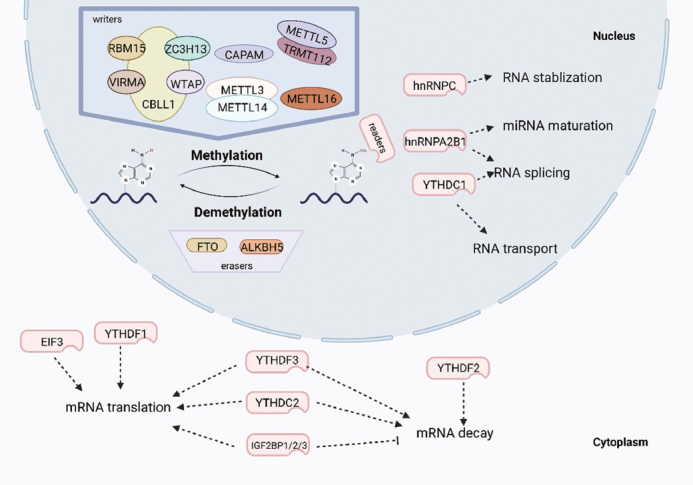
Overview of m^6^A modification. m^6^A RNA methylation is established by ‘writers’, eliminated by ‘erasers’, and identified by ‘readers’.

Over 170 types of RNA modification products exist; these include 5-methylcytidine (m^5^C), N^1^-methyladenosine (m^1^A), and N^4^-acetylcytidine (ac^4^C), which can regulate the structure, function and bioprocessing of RNA.^[12]^ One of the most well-studied products is N^6^-methyladenosine (m^6^A), generated by dynamic epigenetic modification regulated by various factors in which the hydrogen atom of an adenine nucleotide is replaced by a methyl group at the N^6^ position. Clusters of regulators, including “writers” responsible for executing the modification, “erasers” responsible for removing the methylation and “readers” responsible for identifying the modification, work together harmoniously to maintain steady and balanced m^6^A levels (Figure 1).

Since its first discovery in the 1970 s, m^6^A has been identified as the most prevalent mRNA modification in most eukaryotes (including mammals, insects, plants, yeast and some viruses).^[13]^ However, due to the lack of molecular biology, quantitative and sequencing methods to comprehensively study m^6^A modifications in the transcriptome, the field did not make much progress in the following decades. In 2011, fat mass and obesity-associated protein (FTO) was identified as the first m^6^A demethylase, a finding that suggested that m^6^A modification is reversible and dynamic and therefore may have important functions.^[14]^ The modification profile of m^6^A in the transcriptome was first mapped in 2012 by next-generation sequencing (NGS) technology.^[15]^ Currently, m^6^A is found on approximately one-third of mammalian mRNAs, with an average of 3-5 m^6^A modifications per mRNA, and many m^6^A sites are evolutionarily conserved in humans and mice.^[16]^ To date, several antibody-dependent (*e.g*., MeRIP-seq and miCLIP) and non-antibody-dependent (*e.g*., MAZTER-seq, m^6^A-REF-seq and DART-seq) sequencing methods have been developed that make high-resolution detection of m^6^A epitopes and modification composition in different cellular environments a reality^[15,17,18,19,20]^ (MeRIP-seq: methylated RNA immunoprecipitation with next-generation sequencing; miCLIP-seq: m^6^A individual-nucleotide-resolution cross-linking and immunoprecipitation with sequencing; MAZTER-seq: RNA digestion via m^6^A sensitive RNase; m^6^A-REF-seq: m^6^A-sensitive RNA-endoribonuclease-facilitated sequencing; DArT-seq: deamination adjacent to RNA modification targets sequencing).

Next-generation sequencing analysis illustrated that m^6^A modifications consistently occur in the typical motif DRACH. The DRACH motif is a consensus sequence of 5 nucleotides described as [G/A/*U*] [G > A] AC [*U* > A > C], where A could be modified to m^6^A.^[21]^ Despite the ubiquity of DRACH sequences in the transcriptome, only 1%-5% of them are methylated *in vivo*.^[22]^ Notably, m^6^A is not randomly distributed throughout the transcript but is preferentially detected in the coding sequence, 3’-untranslated regions (UTRs), and particularly the regions adjacent to the stop codon.^[16]^ Since m^6^A was recognized 30 years ago, m^6^A-seq, also known as methylated RNA immunoprecipitation sequencing, has revealed the roles of m^6^A in humans and mice and revived the intensive focus on the underlying mechanisms of m^6^A modification and its multiple functions in many aspects.^[23,24]^

The modification associated with m^6^A functions in almost all the major cellular activities in eukaryotic cells; it determines the fate of RNAs, including mRNAs and noncoding RNAs, and consequently plays a significant role in multiple bioprocesses, including normal and pathogenic development.^[25,26,27]^ m^6^A modification has been shown to impact multiple cellular and biological processes, including the development of the nervous system, ovarian aging, spermatogenesis, fertility, sex determination and pluripotency and developmental programs.^[28,29,30,31,32,33,34]^ Aberrant expression of diverse regulators and dysregulation of m^6^A modifications have been reported to be associated with cancers. Usually, abnormal m^6^A methylation is derived from a loss of stable balance between genomic and epigenetic regulation, leading to upregulation (or downregulation) of gene expression, which is associated with sustained proliferation, disrupted apoptosis, abnormal stemness, and treatment failure, leading to cancer cell initiation, progression and drug resistance.^[35,36,37]^ Many oncogenes can act on m^6^A regulators to alter RNA m^6^A methylation levels and contribute to cancer occurrence and development. For example, in hepatocellular carcinoma (HCC), m^6^A modification upregulates stemness through multiple signaling pathways.^[38,39]^ In addition, the expression of regulators such as reader YTH domain-containing 2 (YTHDC2) and eraser fat mass and obesity-associated protein (FTO) has important prognostic value for HCC.^[40]^ Furthermore, m^6^A modification can be employed to prevent radiofrequency ablation (RFA) -induced HCC metastasis and recurrence: for example, it was revealed that m^6^A mechanism-targeted therapy binds to epidermal growth factor receptor (EGFR) inhibitors to prevent HCC metastasis after RFA.^[41]^ Another eraser, AlkB homolog 5 (ALKBH5), is upregulated in epithelial ovarian cancer and induces cancer cell resistance to cisplatin.^[42]^

Recently, there has been increasing interest in the relationship between N^6^-methyladenosine and cancer. A surge in the abundance of m^6^A RNA, particularly mRNA, has been reported in GC tissues compared to adjacent normal control tissues, implying that m^6^A modifications play a key role in GC tumorigenesis and progression.^[43]^ In addition, the dysregulation of m^6^A levels and the levels of m^6^A regulators, such as writers, erasers, and readers, has a substantial impact on tumorigenesis, proliferation, invasion, metastasis, drug resistance, and cancer relapse in GC.

## Writers, erasers, and readers and their cooperation

Interest in the N^6^-methyladenosine modification has exploded over recent years with the discovery of writers, erasers, and readers. The relationship between m^6^A and mRNA has been revealed. m^6^A modification occurs on most transcripts, with the ratio of m^6^A/A in mRNAs ranging from 0.2% to 0.5%.^[44]^ Reversible and dynamic m^6^A modifications are involved in physiological processes such as mRNA transport out of the nucleus, translation and degradation and are also closely correlated with tumor cell proliferation, apoptosis, metastasis and chemotherapy resistance. m^6^A is also prevalent in noncoding RNA. For example, it facilitates tumorigenesis in lung cancer by upregulating the stability of lung cancer associated transcript 3 (LCAT3, a lncRNA).^[45]^ Recent advances in m^6^A modifications of RNA and their biological properties will be reviewed below (Table 1).

**Table 1:** m^6^A regulators and their roles in m^6^A

Regulators	Proteins		Location	Function
Writers	METTL3	Methyltransferase-like 3	Nucleus	Catalytic subunit of m^6^A methyltransferase complex; binding to 3’-UTRs can promote translation independent of m^6^A methylation
	METTL14	Methyltransferase-like 14	Nucleus	RNA-binding scaffold of m^6^A methyltransferase complex, stabilize the structure of the METTL3-METTL14 methyltransferase complex and promote RNA substrate recognition to enhance the methyltransferase activity of METTL3
	WTAP	Wilms’ tumor 1-associating protein	Nucleus	Regulatory subunit of m^6^A methyltransferase complex, maintaining the nuclear localization and nuclear speckle enrichment of the m^6^A writer complex and leading METTL3-METTL14 heterodimer to mRNA
	METTL16	Methyltransferase-like 16	Cytoplasm and nucleus	Independent methyltransferase
	VIRMA/KIAA1429	Vir-like m^6^A methyltransferase associated	Nucleus	Subunit of m^6^A methyltransferase complex, acting as an adaptor protein to lead METTL3-METTL14 heterodimer to mRNAs
	RBM15	RNA binding motifs protein 15	Nucleus	Subunit of m^6^A methyltransferase complex, facilitating recruitment of methyltransferase complex on target RNA
	RBM15B	RNA binding motifs protein 15B	Nucleus	Subunit of m^6^A methyltransferase complex, facilitating recruitment of methyltransferase complex on target RNA
	ZC_3H_13	Zinc finger CCCH domain-containing protein 13	Nucleus	Facilitates methyltransferase complex RNA binding
	CBLL1/HAKAI	Cbl proto-oncogene like 1	Nucleus mainly	Essential for stabilization of m^6^A-METTL associated complex
Erasers	FTO	Fat mass and obesity-associated protein	Nucleus and cytoplasm	Demethylase (catalytic site: H231 and D233) and participate in mRNA splicing
	ALKBH3	AlkB homolog 3	Cytoplasm	demethylase specific to tRNA
	ALKBH5	AlkB homolog 5	Nucleus	Demethylase (catalytic site: H204 or H266), contribute to processing and exporting of mRNA and stabilizing of pre-mRNA in nuclear speckles
Readers	YTHDF1	YTH domain family proteins 1	Cytoplasm	Direct reader of mRNA to promote translation
	YTHDF2	YTH domain family proteins 2	Cytoplasm	Direct reader of mRNA to promote degradation
	YTHDF3	YTH domain family protein 3	Cytoplasm	Works with YTHDF1 and YTHDF2, facilitating mRNA translation and degradation
	YTHDC1	YTH domain-containing protein 1	Nucleus	Direct reader of miRNA that regulates splicing
	YTHDC2	YTH domain-containing protein 2	Cytoplasm	Context-dependently promote mRNA translation or degradation
	eIF3	Eukaryotic initiation factor 3	Cytoplasm	Promotes cap-independent mRNA translation
	IGF2BP1	Insulin-like growth factor 2 mRNA-binding protein 1	Cytoplasm	Increases the stability of mRNA by inhibiting degradation or increasing storage in stress condition and facilitating their translation (probably by recruiting RNA stabilizers)
	IGF2BP2	Insulin-like growth factor 2 mRNA-binding protein 2	Cytoplasm	
	IGF2BP3	Insulin-like growth factor 2 mRNA-binding protein 3	Cytoplasm	
	hnRNPC	Heterogeneous ribonucleoprotein C	Nucleus	Binds and controls processing of nascent RNA
	hnRNPA2B1	Heterogeneous ribonucleoprotein A2B1	Nucleus	Direct reader of miRNA splicing and miRNA maturation

### Writers

The discovery of a methyltransferase complex acting on nascent pre-mRNAs for m^6^A methylation has greatly stimulated interest in m^6^A. Writers are a set of m^6^A methyltransferase complexes through which methyl groups are attached to RNA.^[46]^ m^6^A action on mRNA is catalyzed by the m^6^A-METTL complex (MAC), consisting of methyltransferase-like 3 (METTL3), methyltransferase-like 14 (METTL14), and the m^6^A-METTL-associated complex (MACOM). MACOM is composed of Wilms’ tumor 1-associating protein (WTAP), zinc finger CCCH-type containing 13 (ZC3H13), RNA binding motifs protein 15 (RBM15), Vir-like m^6^A methyltransferase-associated (VIRMA/KIAA1429), and Cbl proto-oncogene like 1 (HAKAI/CBLL1).^[47]^ METTL3 and METTL14 form the heterodimeric complex and, together with CCCH-type zinc fingers, constitute the minimally required regions to exert m^6^A modifications *in vitro*. The N^6^-methylation by METTL3 involves S-adenosylmethionine (SAM) as the methyl donor, and METTL14 is required to enhance METTL3 activity by binding substrate RNA and positioning the methyl group for transfer to adenosine.^[48]^ Most m^6^A sites of the METTL3/METTL14 apparatus are enriched at the 3’ UTRs and near-stop codons.^[15,16]^ WTAP interacts with the METTL3-METTL14 complex, allowing it to localize to nuclear speckles along with pre-mRNA processing agents and function to regulate MAC recruitment to mRNA targets for catalytic activity in vivo. When WTAP is absent, the capacity of METTL3 to bind RNA is greatly reduced.^[49]^

The core complex consists of METTL3, METTL14, and WTAP, and the linker proteins KIAA1429, RBM15, HAKAI, and ZC_3_H_13_ can bind to heteropolymers and function with the core methyltransferase complex to determine the correct location of MAC. Human KIAA1429, the largest known component in the m^6^A methyltransferase complex, contains a C-terminal (C-KIAA1429) and N-terminal (N-KIAA1429) and preferentially mediates mRNA methylation in the 3’UTR and near the stop codon.^[50]^ KIAA1429 might serve as a scaffold linking the METTL3/METTL14/WTAP catalytic core components and RNA substrates and affect the site-specific installation of m^6^A through its N-KIAA1429 domain.^[51]^ ZC_3_H_13_ is an adapter connecting the RNA-binding protein RBM15 to WTAP.^[52]^ AKAI, also called CBLL1, is a conserved member of the MACOM, and its ubiquitination domain is essential for maintaining MACOM integrity. Consistent with its role in the m^6^A pathway, HAKAI plays a role in the sex-determined pathway and mediates the splicing of sexual death. ^[53,54]^

Research into the structure and function of human m^6^A writers is flourishing. Recent studies have identified METTL16, another enzyme, as a novel m^6^A methyltransferase. METTL16 contains N-terminal RNA-binding and methyltransferase and interacts with a multitude of RNAs, including MAT2A mRNA, MALAT1 lncRNA, and U6 snRNA.^[55,56,57]^ It also utilizes SAM as a methyl donor, similar to METTL3.^[57,58]^ Compared with METTL3/METTL14’s RRACH motif, that of METTL16 requires both a UACAGAGAA consensus sequence and a specialized stem–loop RNA structure. METTL16 impacts many m^6^A modifications in the epidermal transcriptome and manages the splicing of SAM synthetase transcripts to guard against SAM homeostasis.^[59]^ The METTL5/TRMT112 heterodimeric complex was recently demonstrated to be a methyltransferase linked to 18S rRNA m^6^A modification, and ZCCHC4 is an enzyme involved in the A4220 modification of 28S rRNA.^[60,61]^ Similar to METTL3/METTL14, METTL5 is the catalytic subunit of the complex, and TRMT112 may be involved in RNA binding and METTL5 activation to stimulate its interaction with SAM. A parallel *β*-zipper links the two proteins, and TRMT112 stabilizes METTL5 by masking a sizeable hydrophobic patch on it.^[60]^ Recently, in eukaryotic mRNA, cap-specific adenosine methyltransferase identified as PCIF1 was found to act on the m^6^A of 2’-O-methyladenosine (Am) to construct the m^7^Gpppm^6^ Am pattern, provided that Am is the first nucleotide transcribed. ^[62,63]^

### Erasers

Erasers are a group of proteins that remove methyl groups from RNA molecules modified with m^6^A, including two types of demethylating enzymes, FTO and ALKBH5. The establishment of erasers makes N^6^-methyladenosine dynamic and reversible. FTO was the first identified m^6^A demethylase and is localized to nuclear speckles and the cytoplasm.^[14]^ It has been established that FTO has competent oxidative demethylation activity against ample m^6^A residues in RNA.^[14]^ In addition to the N^6^-methyladenosine in mRNA, N^6^, 2’-O-dimethyladenosine (m^6^A_m_) of the mRNA and snRNA is also the substrate of FTO, which is relevant to mRNA stabilization by resisting DCP2-mediated mRNA-decapping.^[64,65]^ FTO catalyzes the demethylation of m^6^A only in the nucleus, while it can modulate the demethylation of m^6^A and m^6^A_m_ in the cytoplasm. There are controversies about the affinity of FTO for m^6^A and m^6^A_m_, but most concur that FTO has a higher affinity for m^6^A in the nucleus and a stronger affinity for m^6^A_m_ in the cytoplasm. ^[14,65]^

ALKBH5 is another established protein with m^6^A demethylation activity in mammals.^[66]^ It affects nuclear RNA metabolism, export, and gene expression and plays a broad role in essential processes with its demethylation activity in vivo and *in vitro*.^[66]^ ALKBH5 demethylates the m^6^A-containing ssRNA with activity comparable to that of FTO.^[66]^ While FTO and ALKBH5 belong to the iron- and 2-oxoglutarate-dependent family of AlkB oxygenases, their physiological functions are distinct. For example, FTO appears to be closely linked with obesity, but ALKBH5 has been shown to be essential for spermatogenesis.^[66,67]^ Regarding the expression level, FTO is highly expressed in the brains of mice, while ALKBH5 is highly expressed in the testes. To achieve such biological function differences, FTO and ALKBH5 may specifically catalyze the demethylation of target mRNAs.

### Readers

Proteins that bind to the methylation site of m^6^A are called readers or m^6^A recognition proteins. They selectively recognize m^6^A on target RNA and participate in various metabolic processes of the RNA. Readers include YTHDC1–2 and YTHDF1–3 (YTH domain-containing proteins), IGF2BP1–3 (insulin-like growth factor 2 mRNA-binding proteins), and hnRNPA2B1 and HNRNPC/G (heterogeneous nuclear ribonucleoproteins).

YTHDC1 is located in the nucleus, and YTHDC2 and YTHDF1-3 are located in the cytoplasm.^[68,69,70]^ YTHDC1 binds to and recruits KDM3B, a histone H3 lysine 9 dimethylation (H3K9me2) demethylase, to m^6^A-associated chromatin regions, contributing to H3K9me2 demethylation and promoting gene expression.^[71]^ YTHDC1 modulates splicing, nuclear-cytoplasmic export, and degradation of m^6^A-modified RNAs by regulating splicing factors and nuclear exosome targeting-mediated nuclear degradation.^[68,72,73]^ In contrast, YTHDC2 decreases the stability of m^6^A-modified mRNA by interacting with RNA helicase but increases the translation efficiency of targeted mRNA.^[74,75,76]^

Although they are similar in structure, the functions of YTHDF proteins differ. YTHDF1, YTHDF2, and YTHDF3 form complexes with targeted mRNAs and modulate the stability and translation of YTHDF-bound mRNAs.^[77,78,79]^ YTHDF1 mediates translation facilitation and increases the translation and protein production efficiency of m^6^A-tagged transcripts.^[80]^ In the YTHDF2-mRNA complex, the C-terminal structural domain of YTHDF2 targets m^6^A-mRNA, and the N-terminal structural domain of the complex is responsible for its localization to cellular RNA decay sites.^[79]^ YTHDF3 facilitates protein synthesis via YTHDF1 and mediates methylated mRNA degradation via YTHDF2.^[70]^ All three YTHDF proteins can comprehensively and cooperatively influence fundamental biological processes associated with m^6^A RNA methylation.

IGF2BPs promote RNA stability and increase mRNA storage under dynamic physiological conditions by recruiting RNA stabilizers such as matrin 3. RNA stabilizers also include ELAV-like RNA-binding protein 1 and poly (A) -binding protein cytoplasmic 1.^[81]^

Heterogeneous nuclear ribonucleoproteins (hnRNPs) play a variety of roles in the regulation of transcriptional and posttranscriptional gene expression-related processes, including RNA splicing, modification, translation and degradation.^[82]^ Alarcon *et al*. demonstrated that hnRNPA2B1 could act as a nucleic reader of m^6^A modification and facilitate the processing of a set of METTL3-dependent pri-miRNAs.^[83]^ hnRNPA2B1 can bind G (m^6^A) C-containing nuclear RNAs in vivo and *in vitro*, recruit the microprocessor Drosha-DGCR8 (DiGeorge Syndrome Critical Region 8) complex, and protect its RNA target sites from ribonuclease degradation.^[83]^ Li *et al*. showed that METTL3-triggered LINC01 833 m^6^A methylation promotes non-small cell lung cancer progression through the regulation of hnRNPA2B1.^[84]^ Wu *et al*. suggested that m^6^A can facilitate the ability of hnRNPA2B1 to enhance nuclear events such as pri-miRNA processing by increasing the accessibility of hnRNPA2B1 to certain binding sites instead of facilitating direct binding to m^6^A.^[82]^ Further investigations are required to uncover the details of this mechanism of m^6^A.

### The cooperation of writers, erasers, and readers

The dynamic balance between the deposition and clearance of m^6^A modifications is essential for normal biological processes and development. Mutations and extracellular irritants that induce an increase or decrease in the number of m^6^A modification sites may also affect intracellular levels of m^6^A modification. RNA can be modified by methylation and demethylation through methyltransferases and demethylases, respectively, to maintain appropriate m^6^A and gene expression levels in human tissues and cells. Thus, mutations or dysregulation of writers and erasers are commonly associated with diseases such as cancer, as mutations result in abnormal increases or decreases in m^6^A in RNA transcripts with critical biological functions. Writers and erasers of m^6^A are located in the nucleus and are associated with mRNA splicing factors, suggesting that m^6^A is functionally related to mRNA splicing. ^[14,49,66,85]^ m^6^A can be deposited on RNA transcripts during transcription and affect gene expression post-transcriptionally by altering the structure of the RNA or by specific recognition by readers. YTHDC1 recruits the splicing factor SRSF3 and affects the export of m^6^A-modified mRNA transcription products from the nucleus to the cytoplasm. ^[72,86]^ YTHDFs tend to accelerate the metabolism of m^6^A-modified mRNAs in the cytoplasm. The IGF2BP family protects m^6^A-modified mRNAs in P-bodies and stress granules from degradation and promotes mRNA translation by interacting with ELAV-like RNA binding protein 1 (ELAVL1, also known as Hur), MATR3 (Matrin 3) and poly (A) binding protein cytoplasmic 1 (PABPC1).^[81]^ The m^6^A-modified mRNA acts as a barrier to delay tRNA regulation during translation elongation, thereby disrupting translation elongation kinetics.^[87]^ METTL3 also acts as a m^6^A-binding protein in the cytoplasm, promoting the translation of m^6^A-modified mRNA independent of its methyltransferase activity.^[88,89]^

## Roles of m^6^A in the tumorigenesis and development of GC

**Figure 2:**
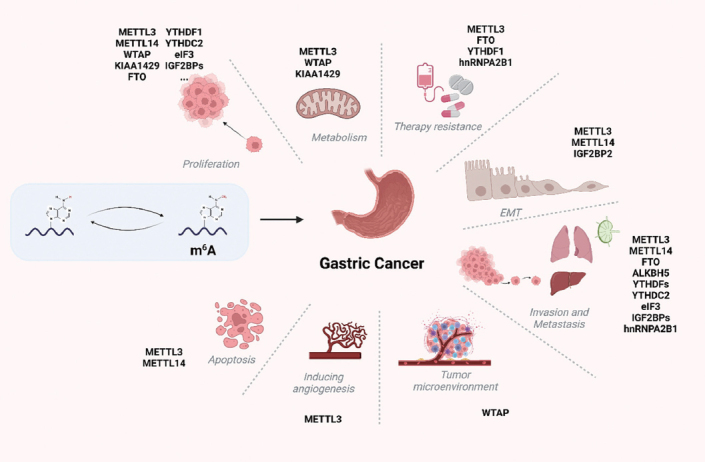
The possible role of RNA m^6^A in GC. m^6^A regulates the expression of oncogenes and tumor suppressor genes and has implications in various processes in GC, including proliferation, metastasis, epithelial-mesenchymal transition, and chemoresistance, and could be used to predict prognosis.

As mentioned above, m^6^A is involved in a range of aspects of cancer. Research on the roles of m^6^A in cancer has progressed considerably, and it is well established that m^6^A has a role in almost all cancer-related processes, including tumorigenesis, proliferation, and remodeling of the tumor microenvironment (TME), angiogenesis, metastasis, immune escape, and chemoresistance. N^6^-methylation not only alters the methylation level of GC cells but also plays diverse roles in GC through its associated regulatory proteins, which play diverse roles in carcinogenesis and progression. Here, we summarize the literature highlighting the significance of m^6^A in the tumorigenesis and progression of GC, as shown in Table 2 and Figure 2.

**Table 2:** m^6^A RNA methylation in GC

Regulator	Up/Down	Mechanism	Phenotype	PMID	Ref.
METTL3	up	promote GFI-1 mRNA expression	Proliferation and migration, EMT	31232471	[90]
		HBXIP/METTL3/m^6^A/MYC	Proliferation, migration and invasion	33048840	[91]
		HOXA10/TGFB2/Smad/METTL3/m^6^A/EMT	Progression and metastasis	33563300	[92]
		stabilize ARHGAP5 mRNA in ARHGAP5-AS1/ARHGAP5 axis	Chemoresistance	31097692	[93]
		PP2Aca/ATM/METTL3	Proliferation	34485508	[94]
		METTL3/SOCS2	Proliferation	32782536	[95]
		LINC00470/METTL3/PTEN	Proliferation, migration and invasion	31711642	[96]
		lncRNA-BLACAT2/miR-193b-5p/METTL3	Proliferation	33976730	[97]
		METTL3/m^6^A/BATF2/p53/ERK	Proliferation and metastasis	32650804	[98]
		METTL3/m^6^A/YAP1	Proliferation and metastasis	34394353	[99]
		EED/miR-338-5p/METTL3/m^6^A/CDCP1	Proliferation and invasion	33882457	[100]
		METTL3/m^6^A/MYC	Proliferation, migration and invasion	33882457	[100]
		METTL3/m^6^A/ZMYM1/E-cadherin	EMT and metastasis	31607270	[101]
		P30/H3K27ac/METTL3/m^6^A/HDGF/GLUT4+EN_O_2	Tumor angiogenesis and glycolysis	31582403	[43]
		METTL3/m^6^A/DGCR8/miR-17-92/AKT/mTOR pathway	Proliferation and metastasis	33037176	[102]
		METTL3/AKT/p70S6K/Cyclin D1	Proliferation, migration and invasion	30886897	[103]
		miR-1269b/METTL3	Inhibit multiplication, migration and invasion	33818282	[104]
		METTL3/m^6^A-YTHDF1/SPHK2/KLF2	Proliferation, migration and invasion	33758320	[105]
		miR-4429/METTL3/m^6^A/SEC62	Inhibit proliferation	31395342	[106]
		SP1/METTL3/THAP7-AS1/CUL4B protein/miR-22-3p and miR-320a	Proliferation, migration and invasion	34608273	[107]
		METTL3/PBX1/GCH1 axis	Proliferation and lung/Lymph node metastasis	35261206	[108]
		METTL3/m^6^A/PARP1	Oxaliplatin resistance	35179655	[109]
		LncRNA LINC02253/METTL3/m^6^A/KRT18/MAPK/ ERK	Proliferation, migration and invasion	35136989	[110]
		LINC00240/miR-338-5p/METTL3	Proliferation, migration and inhibit cell apoptosis	34842045	[111]
METTL14	down	METTL14/LINC01320/miR-495-5p/RAB19	Proliferation, migration, and invasion; aggressive phenotype	34288797	[112]
		METTL14/PI3K/AKT/mTOR	Suppress GC cell proliferation and enhance apoptosis, EMT	33314339	[113]
		METTL14/m^6^A/circORC5/miR-30c-2-3p/AKT1S1	Suppress proliferation and invasion	35164771	[114]
WTAP	up	T-cell-related immune response	Tumor immunosuppression	32176425	[115]
		Enhances the stability of HK2 mRNA and promotes Warburg effect of GC cells	Tumor progression	33378974	[116]
METTL16	up	METTL16/m^6^A/cyclin D1	Proliferation and colony formation	34075693	[117]
KIAA1429	up	KIAA1429/m^6^A/c-Jun	Proliferation	32052427	[118]
		KIAA1429/m^6^A/LINC00958/GLUT1	Promote aerobic glycolysis	34409730	[119]
FTO	up	FTO/m^6^A/mTORC1/DDIT3	Chemosensitivity	33393595	[120]
		FTO/HOXB13/IGF-1R/PI3K/AKT/mTOR	Proliferation, migration, and invasion	33894267	[121]
		FTO/m^6^A/ITGB1	Metastasis	34277426	[122]
		HDAC3/FOXA2/FTO/m^6^A/MYC	Promotes viability, migration and invasion	32655129	[123]
		FTO/m^6^A/caveolin-1	Proliferation, migration, invasion and metastasis	35064107	[124]
ALKBH5	up	ALKBH5/ZNF333/CYLD/CDX2	Intestinal metaplasia development	34631277	[125]
		ALKBH5/m^6^A/LncRNA NEAT1/ EZH2	Invasion and metastasis	31290116	[126]
		ALKBH5/PKMYT1/IGF2BP3	Invasion and metastasis	35114989	[127]
YTHDF1	up	YTHDF1/m^6^A/USP14	Proliferation, invasion, gastric tumorigenesis and lung metastasis	33791305	[128]
		YTHDF1/m^6^A/FZD7/Wnt/β-catenin	Proliferation and carcinogenesis	32788173	[129]
		YTHDF1/IFN-Y receptor 1 and JAK/STAT1	Sensitivity to antitumor immunity	35193930	[130]
YTHDF2	down	YTHDF2/m^6^A/FOXC2	Inhibit proliferation, invasion and migration	33505426	[131]
YTHDF3	up	sEV-miR-151a-3p/miR-151a-3p/YTHDF3/m^6^A/SUMO1/SP3/TGF-β1/SMAD (2/3)	Liver metastasis	34535770	[132]
YTHDC2	up	YTHDC2/m^6^A/YAP	Proliferation, migration and invasion	34911015	[133]
eIF3	up	EIF3B/PI3K/AKT/mTOR	Proliferation, migration and invasion	31686906	[134]
IGF2BP1	up	lncRNA TRPM2-AS/miR-612/IGF2BP1	Progression and metastasis	32123162	[135]
		lncRNA GLCC1/c-Myc/IGF2BP1	Proliferation, apoptosis, migration and invasion	34196212	[136]
IGF2BP2	up	LINC01559/IGF2BP2/ZEB1	Proliferation, migration and EMT	33824282	[137]
		IGF2BP2/IGF1R/RhoA/ROCK	Carcinogenesis	35306138	[138]
IGF2BP3	up	microRNA-125a-5p/IGF2BP3	Proliferation	32266868	[139]
		circFNDC3B/IGF2BP3/CD44	Migration and invasion	30963578	[140]
		H19-PEG10/IGF2BP3	Proliferation and invasion	29088808	[141]
		miR-34a/IGF2BP3	Tumorigenesis	28399871	[142]
		circ-TNPO3/IGF2BP3/MYC/SNAI	Proliferation and metastasis	34703650	[143]
hnRNPA2B1	up	hnRNPA2B1/BIRC5	Proliferation, metastasis and chemoresistance	34044823	[144]

### Tumorigenesis and proliferation

m^6^A has been discovered to be associated with the tumorigenesis of GC, but the precise mechanism is unclear. Both METTL3 and METTL14 belong to the m^6^A methyltransferase complex, but they have different roles in GC. METTL3 was reported to promote the proliferation and migration of GC cells via the m^6^A modification of YAP1.^[99]^ METTL3 knockdown inhibits cell proliferation, migration, and invasion in GC cells.^[103]^ Another study showed that embryonic ectoderm development (EED) promotes GC development by downregulating miR-338-5p through histone methylation, thereby impairing miR-338-5p-dependent METTL3 inhibition and enhancing Cub domain containing protein 1 (CDCP1) translation.^[100]^ METTL14-mediated m^6^A modification leads to upregulation of LINC01320, which promotes the proliferation and invasion of GC cells, with LINC01320 knockdown exerting a deleterious effect.^[112]^ Conversely, the overexpression of METTL14 restrains GC cell proliferation by inhibiting the PI3K/AKT/mTOR pathway (PI3K, phosphatidylinositol 3-kinase; AKT, serine/threonine kinase; mTOR, mammalian target of rapamycin) and suppresses invasion by affecting the epithelial-mesenchymal transformation (EMT) pathway.^[113]^ As for other writers, METTL16 exerts a pro-oncogenic effect by enhancing the stability of cyclin D1 mRNA in GC cells.^[117]^ KIAA1429 could act as an oncogenic factor in GC by stabilizing c-Jun mRNA in an m^6^A-independent manner.^[118]^

Erasers also play a facilitating role in GC. FTO restrains HOXB13 methylation, and the overexpression of FTO and HOXB13 drives GC cell proliferation, migration, and invasion through PI3K/AKT/mTOR signaling via IGF-1R.^[121]^ Moreover, FTO demethylates caveolin-1 mRNA, enhances its degradation, regulates mitochondrial metabolism, and promotes cell proliferation and metastasis in GC.^[124]^ Yue *et al*. suggested that a positive feedforward loop between ALKBH5 and NF-*ϰ*B signaling associated with m^6^A modification generates the intestinal metaplasia phenotype of gastric epithelial cells.^[125]^

Most readers also play oncogenic roles in GC through various pathways. Among the protein family containing the YTH domain, YTHDF1 plays the opposite role to YTHDF2 in GC development. YTHDF1 promotes the translation of frizzled 7 (FZD7) in an m^6^A-dependent manner and enhances overactivation of the Wnt/β-catenin pathway, promoting carcinogenesis.^[129]^ The orthodox Wnt signaling pathway plays a key role in the regulation of proliferation, stem cell maintenance and homeostasis in normal gastric mucosa, in addition to self-renewal of GC stem cells.^[145,146,147]^ The dysregulation of the Wnt pathway participates in the development of human cancers and Wnt/β-catenin pathway genes are found among those affected by dysregulation of miRNAs in many kinds of cancers.^[145,148]^ Activation of Wnt/*β*-catenin signaling can be found in over 30% of GCs and is involved in many miRNA- and lncRNA-related GC pathways.^[145,149]^ YTHDF2 has lower expression in GC tissues and cells, regulates the stability of Forkhead box protein C2 (FOXC2) mRNA and inhibits the proliferation and migration of GC cells.^[131]^ For other readers without a YTH structural domain, IGF2BPs play a carcinogenic role in GC tumorigenesis and proliferation. Yang *et al*. demonstrated that glycolysis-associated lncRNA of colorectal cancer (GLCC1) mediates GC cell migration and invasion by fostering the c-Myc/IGF2BP1 interaction.^[136]^ IGF2BP2 is recruited to and binds to LINC01559 to stabilize zinc finger E-box binding homeobox 1 (ZEB1) mRNA and promotes GC progression.^[137]^ IGF2BP3 is an essential target of miR-34a in gastric carcinogenesis and is upregulated in the presence of miR-34a silencing. IGF2BP3 knockdown significantly inhibits cell proliferation and invasion.^[142]^ In addition to the YTHDF and IGF2BP protein families, EIF3B is strongly associated with proliferating cell nuclear antigen expression and PI3K/AKT/mTOR pathway activity in GC samples.^[134]^ hnRNPA2B1 controls the selective splicing of the antiapoptotic factor BIRC5, which promotes cell proliferation, inhibits apoptosis and enhances cell metastasis in GC, and its overexpression is associated with low survival.^[144]^

### Epithelial-mesenchymal transition and metastasis

Tumor metastasis is a complicated process and the main factor affecting the treatment and prognosis of malignant disease. Tumor metastasis involves a variety of neoplastic behaviors and is closely correlated with poor prognosis. The EMT, migration and invasion of cancer cells into surrounding tissues are all closely related to tumor metastasis.

METTL3 is required for the EMT process *in vitro* and for metastasis *in vivo*.^[90]^ Zinc finger MYM-type containing 1 (ZMYM1) is a well-established m^6^A target of METTL3, and ZMYM1 mRNA is stabilized by METTL3-induced m^6^A modification. ZMYM1 facilitates EMT and metastasis by restraining the E-cadherin promoter by promoting the CtBP/LSD1/CoREST complex (C-terminal binding protein, CtBP; histone demethylase, LSD1; corepressor of RE1 silencing transcription factor, CoREST).^[101]^ It has also been reported that METTL3 binds and stabilizes pre-B-cell leukemia homeobox 1 (PBX1) mRNA to induce further expression of GTP cyclohydrolase 1 (GCH1), thereby increasing the level of tetrahydrobiopterin (BH4) in GC cells and promoting tumor progression and lung/Lymph node metastasis.^[108]^ In contrast, overexpression of METTL14 suppresses the growth and invasion of GC cells *in vitro*. METTL14 may mediate the activity of the PI3K/AKT/mTOR pathway by increasing the levels of phosphorylated PI3K, AKT, and mTOR proteins, which are essential for cell proliferation and development.^[113]^ In addition, METTL14 downregulation increases the levels of vimentin, N-cadherin, and matrix metalloproteinase 9 protein and decreases the expression of E-cadherin protein, suggesting that METTL14 overexpression could inactivate the EMT pathway.^[113]^

In addition to the above writers, FTO is confirmed as an independent risk factor for predicting the overall survival (OS) of GC.^[150]^ FTO promotes GC metastasis by upregulating the expression of Integrin b1 (ITGB1) through demethylation.^[122]^ ALKBH5, another demethylase, influences the expression of lncRNA nuclear paraspeckle assembly transcript 1 (NEAT1), and the overexpression of NEAT1 leads to overexpression of enhancer of zeste homolog 2 (EZH_2_), a subunit of the polycomb repressive complex, which subsequently affects GC invasion and metastasis.^[126]^

The proteins responsible for demethylation are also involved in tumor metastasis. YTHDF1 is thought to facilitate GC tumorigenesis and metastasis in an m^6^A-dependent way by promoting ubiquitin-specific protease 14 protein translation.^[128]^ YTHDF3 suppresses small ubiquitin-related modifier SUMO1 translation in an m^6^A-dependent way in Kupffer cells, participates in the inhibition of SP3 processing by sEV-miR-151A-3p, and thus accelerates liver metastasis in GC.^[132]^ As another member of the YTH family, YTHDF2 prohibits GC proliferation and migration by destabilizing FOXC2 mRNA, and its overexpression significantly reduces protein expression in the FOXC2 signaling pathway.^[131]^ The proteins of the IGF2BP family also play an active role in the development of GC, and this role is partially associated with lncRNAs or circRNAs. The interaction of IGF2BP1 protein and c-Myc mRNA is enhanced by the upregulation of lncRNA GLCC1, which promotes the stabilization of c-Myc mRNA, and its knockdown contributes to apoptosis in GC cells.^[136]^ IGF2BP3 increases GC migration and invasion via the formation of a ternary complex of circFNDC3B-IGF2BP3-CD44 mRNA.^[140]^

### Therapeutic resistance

The treatments for GC mainly include surgery, systemic chemotherapy, radiotherapy, and immunotherapy, which have proven efficacy in GC.^[7]^ Resistance to multiple therapies due to various genetic and epigenetic variations remains the biggest obstacle to the treatment of GC.

In terms of chemical therapy, ARHGAP5-AS1 was identified as an upregulated lncRNA in chemo-resistant GC cells that stabilizes ARHGAP5 mRNA by promoting m^6^A modification of ARHGAP5 mRNA through recruitment of METTL3.^[93]^ The upregulation of ARHGAP5 promotes chemotherapy resistance and predicts a poor prognosis in GC.^[93]^ Oxaliplatin is the first-line treatment for advanced GC,^[151]^ and poly (ADP-ribose) polymerase 1 (PARP1) is the crucial gene generating oxaliplatin-resistant hallmarks in CD133^+^ GC stem cells by efficiently repairing DNA damage caused by oxaliplatin. The increased levels of m^6^A mRNA and its writer METTL3 stabilize PARP1 by mobilizing YTHDF1 to the 3’-untranslated region of PARP1 mRNA and promote resistance to oxaliplatin *in vitro*.^[109]^ In addition, a phase II study demonstrated the efficacy of everolimus in previously treated patients with advanced GC.^[152]^ Recent studies have found that everolimus can improve the chemosensitivity of GC by targeting the METTL3/miR-17-92/TMEM127 or PTEN/ mTOR signaling pathways. Further studies showed that GC cells with high METTL3 expression are more sensitive to the mTOR inhibitor everolimus, which could reverse METTL3-induced tumor proliferation in a dose-dependent manner.^[102]^ Feng *et al*. found that m^6^A modification and its eraser FTO may play a role in omeprazole-mediated improvement of chemosensitivity.^[120]^ Omeprazole-induced FTO inhibition enhances the activation of the mTORC1 signaling pathway and suppresses survival-friendly autophagy, thereby improving the antitumor effects of chemotherapeutic agents on GC cells.^[120]^

Concerning the immune microenvironment, YTHDF1 inhibits the recruitment of mature dendritic cells (DCs) in GC and suppresses antitumor immunity.^[153]^ YTHDF1 deficiency mediates the upregulation of the JAK/STAT1 (Janus kinase/signal transducer and activator of transcription) pathway and promotes the expression of IFN-*γ* receptor 1, thereby enhancing antitumor immunity.^[130]^ The loss of YTHDF1 induces persistent systemic antitumor immunity, and YTHDF1 may be highlighted as a possible therapeutic prospect in GC.

## Clinical application of m^6^A in GC

**Figure 3:**
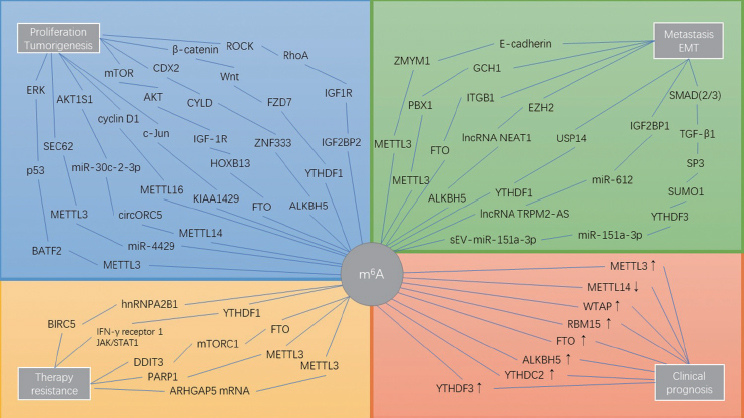
m^6^A modification alters the expression of oncogenes and tumor suppressor genes, which contributes to processes involved in the development of GC, including proliferation, metastasis, epithelial-mesenchymal transition, and chemotherapy resistance and also affects prognosis.

The m^6^A methylation modifications are regulated by writers, erasers and readers, and alterations in the expression of the above component genes will cause changes in mRNA expression levels, leading to the occurrence, development and invasion of tumors. Therefore, modulators or inhibitors of m^6^A methylation may be potential therapeutic strategies for malignancies. Due to a series of changes in the methylation level and regulatory protein in GC, assessment of N^6^-methylation levels and regulatory protein expression levels may help in the clinical diagnosis and prognosis evaluation of GC, as shown in Figure 3.

## M^6^A as a diagnostic and prognostic biomarker for GC

Studies are emerging on m^6^A levels and m^6^A-related protein expression as biomarkers for the diagnosis of GC. Ge *et al*. analyzed the levels of m^6^A in total RNA and the expression of associated proteins and showed a surge in GC patients compared to benign and healthy control groups.^[154]^ In addition, the levels of m^6^A and related proteins increase with the malignant progression of GC and decrease in postoperative patients.^[154]^

The expression of the m6A writers METTL3, RBM15 and WTAP is correlated with the pathologic stage.^[111,155,156,157]^ Elevated WTAP levels predict lower recurrence-free survival rates in GC patients.^[157]^ The m^6^A eraser FTO is involved in tumor staging, and ALKBH5 expression is linked to the prognosis of GC. Exceptional high expression of FTO and ALKBH1 mRNA is related to adverse survival rates. A low ALKBH1 protein level is associated with more advanced TNM stages and larger tumor volume, and low FTO expression is correlated with shorter OS in GC patients.^[158]^ Another study also proved that FTO overexpression is an independently valid predictor of prognosis and survival in GC patients.^[157]^ The expression of the m^6^A reader YTHDF3 correlates with tumor stage, and YTHDC2 correlates with the survival of GC patients.^[155]^ Wang *et al*. found that IGF2BP1 single-nucleotide polymorphism is correlated with the risk of discovery stage in GC. The higher the level of IGF2BP1 mRNA expression is, the more prominent and poorer the OS of GC patients.^[159]^

Extensive reports prove the unquestionable importance of lncRNAs in cancer invasion and clinical prognosis because lncRNAs play essential roles in cancer immunity, chromatin modification and transcriptional regulation.^[81,160]^ Wang *et al*. reported an 11-lncRNA signature as a prognostic factor for GC, and the lncRNA signature may contribute to developing personalized immunotherapy therapies.^[81,161]^ Recent studies have investigated N^6^-methyladenosine-related lncRNAs and found that abnormal expression of lncRNAs is a diagnostic and prognostic marker in cancers.^[162]^ Han *et al*. built an m^6^A-related lncRNA prognostic signature (m^6^A-LPS) containing nine hub lncRNAs for GC. Among the studied lncRNAs, the lncRNA AC026691.1 inhibits GC proliferation and migration by upregulating FTO.^[163]^ Another similar study found that an m^6^A-LPS containing 25 lncRNAs could identify individuals at high risk of a poor prognosis. Further research found that resting DCs, monocytes, and memory CD4^+^ T cells are positively correlated with risk markers,^[164]^ which is consistent with primary studies. ^[165,166]^ Moreover, N-cadherin and vimentin, known as biomarkers of EMT, were highly expressed in samples from the high-risk group. These studies provide a new orientation for personalized strategies.

## Therapeutic potential of m^6^A in the treatment of GC

METTL3 is one of the most widely studied regulatory proteins in the progression of methylation and GC, and it is involved in various behaviors, including drug resistance. Oxaliplatin resistance is a significant issue that hinders its therapeutic effect. As mentioned above, METTL3 promotes oxaliplatin resistance in GC stem cells by stabilizing PARP1 mRNA and increasing the activity of the base excision repair pathway.^[109]^ Another published study also showed that METTL3 levels may be a potential predictor of everolimus treatment for GC.^[102]^ These results suggest that downregulation of METTL3 expression by inhibition can contribute to the treatment of GC. Eltrombopag has been discovered to be an inhibitor of METTL3-14 allosterism in acute myeloid leukemia (AML) cells and might be used in antineoplastic therapy.^[167]^ Emerging evidence reveals the association between dysregulation of lncRNAs and chemoresistance via m^6^A.^[168,169]^ LINC00942 stabilizes c-Myc mRNA in an m^6^A-dependent way, and the disruption of the LINC00942-MSI2-c-Myc axis may be a patent therapeutic tool for chemoresistant GC cells.^[170]^

Immunotherapy is one of the most promising anticancer therapies at present. m^6^A modification is potentially associated with immunotherapy characteristics and interferon signal transduction. The tumor mutation burden (TMB) level and microsatellite instability (MSI) ratio are significantly increased in patients with flat expression of the eraser signature, implying that m^6^A modification is relevant to TMB/MSI status and participates in immune responses in GC.^[171]^ m^6^A may also mediate immune responses of GC by modulating the production of interferons, which exert two-tier functions in regulating cancer immunity. Interferons can promote resistance to natural killer cells and limit the effectiveness of antitumor T cells by upregulating PD-L1. ^[171,172]^ Moreover, the knockdown of YTHDF1 enhances the therapeutic effect of PD-L1 checkpoint blockers, suggesting YTHDF1 as a potential target for tumor immunotherapy.^[153]^ In addition, KIAA1429 mediates m^6^A methylation modification and may promote the activation of TME dendritic cells, thus enhancing the antitumor immune response.^[173]^

## Discussion

Due to the clarification of the mechanism of RNA m^6^A modification based on the recent developments in epitranscriptomic studies, its research direction has been gradually extended to various malignancies, including GC. Guan *et al*. constructed an m^6^A score model with diagnostic value for GC using The Cancer Genome Atlas (TCGA) database with high specificity and sensitivity (AUC = 0.986), but the results await large-scale clinical validation.^[157,174]^ Recent studies have demonstrated the diagnostic value of significant dysregulation of m^6^A levels in peripheral blood in malignancies. Zhang *et al*. developed a serum diagnostic marker based on m^6^A target miRNAs for the large-scale detection of cancer. It showed satisfactory sensitivity in identifying GC, and its diagnostic performance was unaffected by sex, age and benign disease.^[175]^ The combination of m^6^A levels in peripheral blood RNA with CEA and CA199 yielded a higher area under the curve in the diagnosis of GC than m^6^A levels alone.^[154]^

Targeted therapy and immunotherapy have an integral role in advanced metastatic GC. As m^6^A modifications are closely associated with tumor resistance, the selection of appropriate m^6^A modulators, inhibitors and activators can improve the effectiveness of GC therapy. The most extensively used inhibitors in clinical practice are FTO inhibitors. Three FTO inhibitors have shown strong anticancer potential. Rhubarb acid was the first identified FTO inhibitor that helps overcome tumor resistance to methylated anticancer drugs.^[176]^ The ethyl ester form of meclofenamic acid inhibits the growth of glioblastoma stem cells.^[177,178]^ R-2-hydroxyglutarate (R-2HG) inhibits the proliferation of leukemia cells.^[179]^ The study of m^6^A modifications in GC provides new insights into the molecular treatment of tumors.

The exploration of m^6^A modification in GC represents a new frontier in cancer research and has gained momentum in recent years, but there are still challenges that need to be addressed. First, many current m^6^A-based scoring systems or predictive models have been obtained using bioinformatics methods or basic experiments, with results awaiting large-scale clinical validation. Datasets of the m^6^A methylome and expression profiles derived from high-throughput analysis are still lacking. Second, although NGS technologies have provided the impetus for advances in the field of m^6^A, the detection methods for m^6^A are more cumbersome and require higher costs, limiting the progress of m^6^A research as well as clinical applications. It remains difficult to accurately identify and locate valuable m^6^A loci. Third, there are few methods for detecting m^6^A sites in noncoding RNAs. Fourth, how m^6^A, an important RNA epigenetic modification, acts in conjunction with DNA and histone epigenetic modifications to regulate gene expression remains to be revealed. Further research is needed on the performance of m^6^A markers in the diagnosis of early-stage cancer.

To address these issues, there is a need to first upgrade existing detection methods and develop new detection methods to address the relative complexity of the detection process and the difficulty of quantifying the levels of complex methylation modifications. Improving the efficiency of m^6^A assays in terms of research targets and equipment or combining these assays with other research methods may be a new avenue. Second, the investigation of the interaction of m^6^A with known GC mechanisms or tumor hallmarks may be more valuable in gaining a deeper understanding of the role of methylation in cancer. Finally, the exploration of aspects of methylation relevant to clinical practice still requires extensive trials and large-scale clinical validation.

## Conclusion and perspective

Advances in detection techniques for m^6^A methylation modification have facilitated the substantial progress in understanding its function in tumors. m^6^A methylation is a “double-edged sword”. Excessive modification of some genes may change the RNA splicing and translation ability, leading to the development and progression of malignant tumors, while some genes lacking m^6^A methylation may promote the occurrence and development of tumors. Due to the heterogeneity of tumors, the aberrant expression of the same writers, erasers, and readers may incur different molecular and phenotypic changes by altering genes into oncogenes or suppressor genes. N^6^-methylation regulation plays an important role in GC, which is of great significance not only for the progression of GC but also for prognosis and diagnosis evaluation. The regulatory mechanism of m^6^A modification in the tumorigenesis and progression of GC requires further exploration, and the subsequent methylation-related research direction may involve the treatment of GC by regulating methylation. Restoring desirable levels of m^6^A methylation may be the key to treatment. The discovery of more modulators and competitive antagonists of m^6^A methylation-related enzymes is of great significance for the exploration of precise and effective targeted drugs for m^6^A-based treatment of GC.
